# Chapter 3. Impact of estrogens on hemostasis

**DOI:** 10.3389/fendo.2025.1617731

**Published:** 2025-09-18

**Authors:** Frank Z. Stanczyk, Intira Sriprasert, Shinnisha Chulapongwanich, Jane L. Yang, Franca Fruzzetti

**Affiliations:** ^1^ Dept of Obstetrics & Gynecology, University of Southern California, Keck School of Medicine, Los Angeles, CA, United States; ^2^ Princess Srisavangavadhana College of Medicine, Chulabhorn Royal Academy, Bangkok, Thailand; ^3^ Clinica San Rossore, Viale delle Cascine, Pisa, Italy

**Keywords:** thrombosis, estrogens, combined oral contraceptive, hemostatic markers, hormone therapy, postmenopausal women, coagulation, fibrinolysis

## Abstract

It is well established that estrogens increase the risk of both arterial and venous thrombosis. Abnormally high levels of some coagulation factors combined with a decrease in anticoagulation factors contribute to thrombotic risk. Although estrogens are known to affect multiple hemostatic markers, the exact molecular mechanism of estrogen-induced thrombosis is unclear. However, small changes in these markers with different types, doses, and/or routes of estrogens may increase thrombotic risk. Most studies on the effect of estrogens have been carried out in premenopausal women using combined oral contraceptives (COCs); studies in postmenopausal women using hormone therapy (HT) are scarce. Short-term studies comparing hemostatic parameters in women receiving either ethinyl estradiol (EE) or estradiol (E_2_), each combined with a different progestin, generally show that EE- and E_2_-based COCs have minimal hemostatic effects on most markers and weaker effects on some markers with E2. The novel estrogen estetrol (E_4_), emerging as a promising option for both hormonal contraception and postmenopausal HT, appears to have a neutral hemostatic effect. The increased procoagulant factors and decreased anticoagulatory mechanisms observed with estrogen use have been linked to an increased venous thromboembolism (VTE) risk and have been studied in women using hormonal contraception or HT. In contraceptive studies, it has been shown that estrogen dosage plays a role in VTE risk, as EE increases this risk in a dose-dependent manner. Although some studies suggest that the progestin type in COCs may affect VTE risk, other studies have found no difference in risk between androgenic and non-androgenic progestins. As for the E_4_-based COC, it is currently being evaluated for VTE risk in post-marketing studies. Regarding postmenopausal HT, both the CEE-alone and CEE/MPA arms of the Women’s Health Initiative trial showed an increased risk of VTE. However, the results are mixed regarding the impact of oral E_2_ on VTE risk. Although some data suggest a lesser impact of transdermal HT on this risk, further studies are needed to confirm this finding.

## Introduction

**Figure 1:**
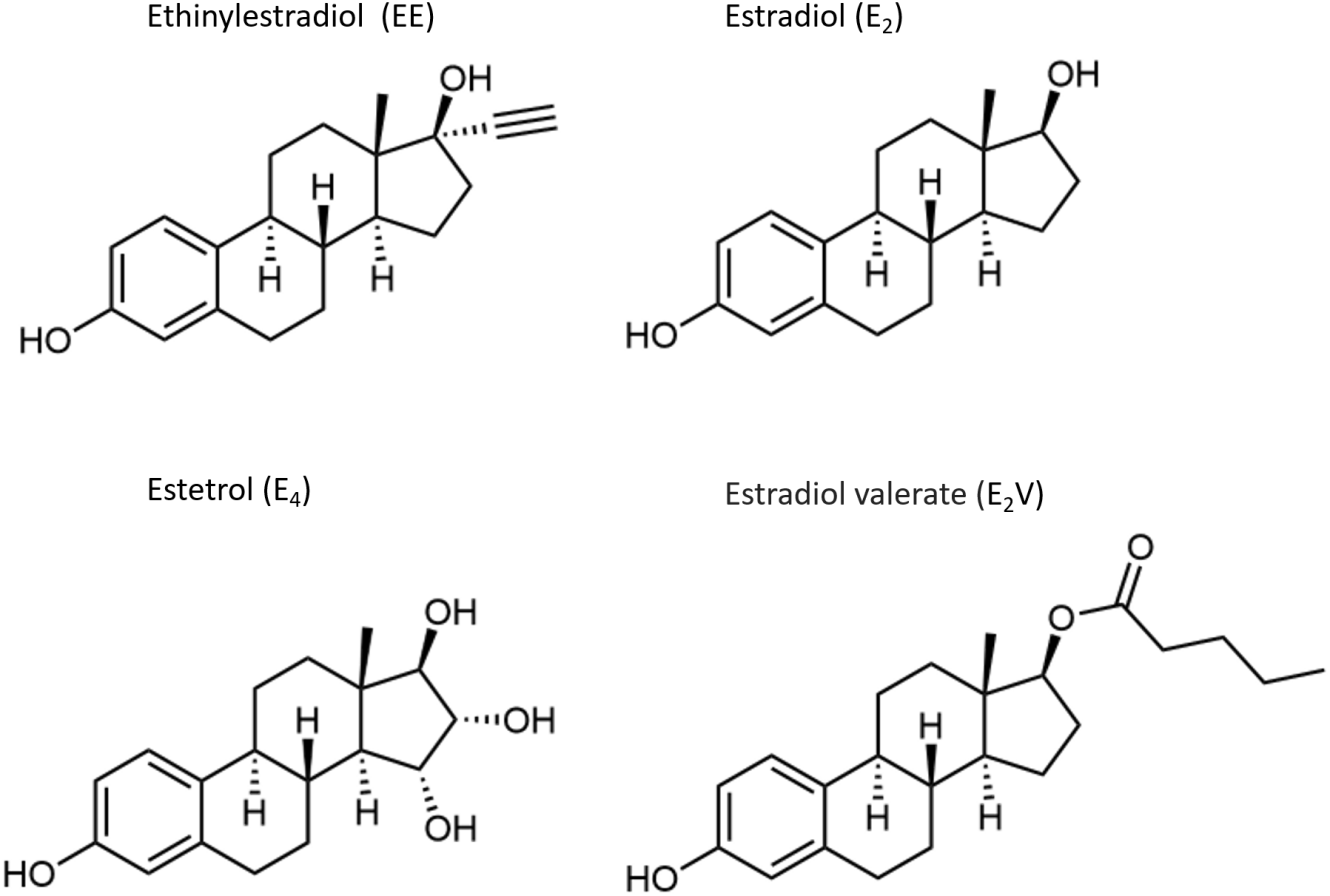
Chemical structures of estrogens.

**Figure 2:**
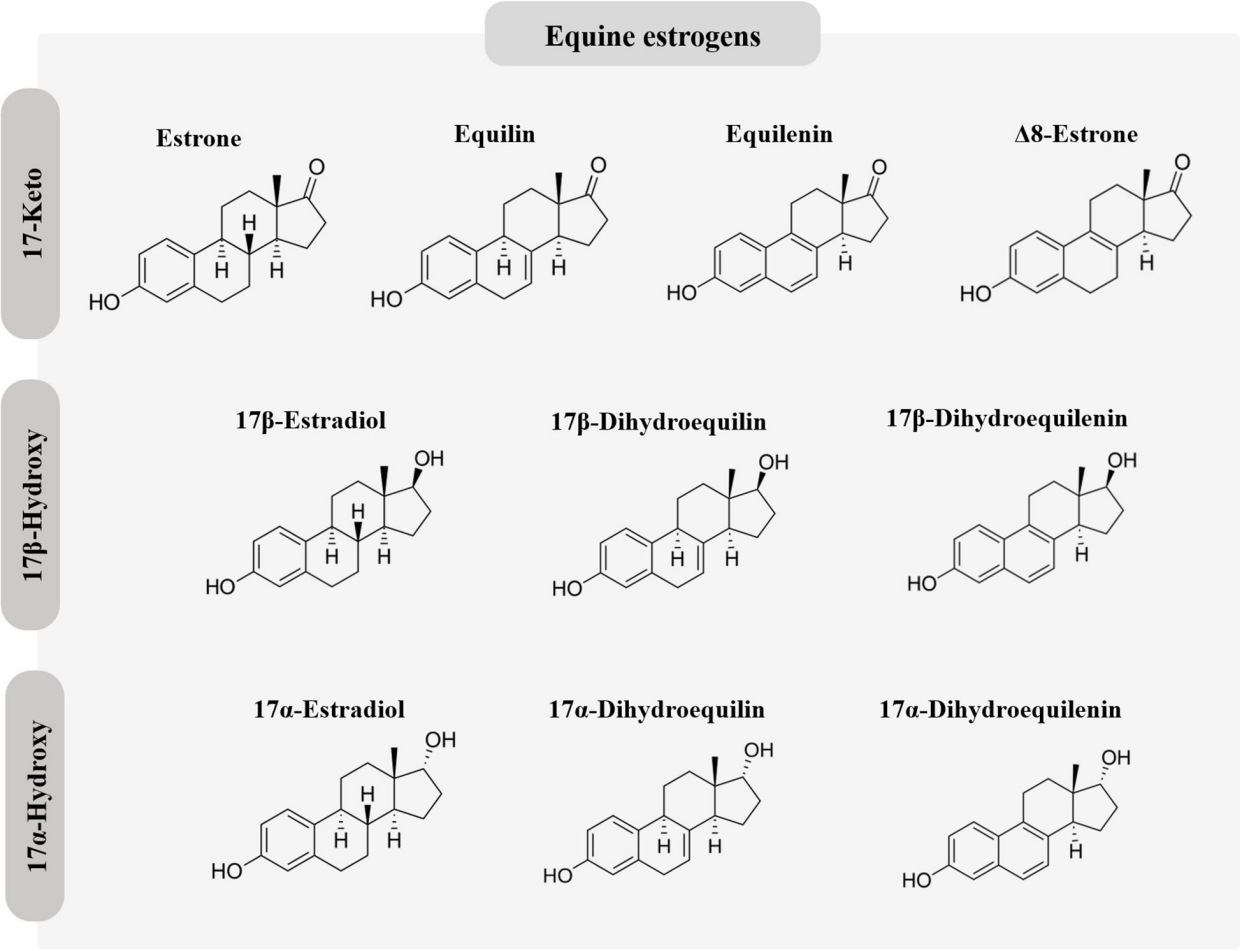
Chemical structures of equine estrogens.

Millions of women worldwide use exogenous estrogens for hormonal contraception or postmenopausal hormone therapy (HT) (1, 2). Estrogen in combination with a progestogen effectively prevents pregnancy by suppressing pituitary gonadotropin release and subsequent ovulation. During menopause, HT is useful for preventing short-term symptoms related to estrogen deficiency as well as long term effects, including changes in bone and cardiovascular health (3). Estrogens used for hormonal contraception generally differ from those used for HT. Ethinyl estradiol (EE) is the predominant estrogen used for hormonal contraception, whereas micronized 17β-estradiol (E_2_) and conjugated equine estrogens (CEEs) are widely used for HT. In recent years, E_2_ and estradiol valerate (E_2_V) have also been used in hormonal contraceptive formulations (4, 5); E_2_V is rapidly converted to E_2_ during the hepatic first pass. Recently, the novel estrogen, estetrol (E_4_), has emerged as a promising option for both hormonal contraception and postmenopausal HT (6). The chemical structures of the estrogens used for hormonal contraception and HT are depicted in **Figures 1** and **2**.

The most common route of administering estrogens is oral, although different estrogen formulations may offer other routes of administration that can be tailored to patients’ individual needs (5). E_2_ can be administered by a variety of different routes, including oral, buccal, sublingual, intranasal, transdermal, vaginal, rectal, intramuscular or subcutaneous injection, and as a subcutaneous implant. CEEs are administered either orally or vaginally. The estrogens are also available in a variety of doses (5).

It is well established that estrogens increase the risk of both arterial and venous thrombosis. Abnormally high levels of some coagulation factors in combination with a decrease in anticoagulation factors contribute to thrombotic risk (7, 8). Following oral administration of estrogen there is a dramatic increase in estrogen-sensitive hepatic proteins, as the highly concentrated estrogen in splanchnic blood is presented to hepatocytes during the hepatic first pass. A variety of proteins are altered, including markers of coagulation, anticoagulation, and fibrinolysis. The exact mechanism by which this occurs is complex and is not understood entirely.

The purpose of the present chapter is to show how different estrogens impact hemostasis in premenopausal and postmenopausal women. We identified articles included in this review by searching in PubMed databases using the following search terms: “estrogens”, “estradiol”, “hemostasis”, “thromboembolism”, and “venous thromboembolism”; all studies were in English.

## Mechanism of Action of Estrogens

Estrogens mediate physiologic processes by genomic and nongenomic mechanisms. Both mechanisms involve the binding of estrogens to estrogen receptors (ERs). There are 2 primary ERs, ERα and ERβ, encoded by different genes; ERα is encoded by the *ESR1* gene on chromosome 6, whereas ERβ is encoded by the *ESR2* gene on chromosome 14 (9). The 2 ERs share common structural components, including DNA binding, ligand-binding, N-terminal, and C-terminal domains. The 2 isoforms vary predominantly in their N-terminal domains and ligand-binding domains. Both ERs are expressed in a wide range of tissues in the body, though their distributions vary across different tissue types and change throughout the lifespan. Recently, a third type of ER has been gaining considerable interest, namely, the G protein coupled estrogen receptor (GPER) (10). In contrast to ERα and ERβ, GPER is a plasma membrane receptor. The physiologic effects of GPER activity are still under study.

The genomic (or classical) mechanism of estrogen action refers to the slow estrogen pathway that takes place over several hours or days (11). In this pathway, estrogen diffuses across the cell membrane into the cytoplasm where it binds to the ERs. The ER complex undergoes a series of coordinated steps that include phosphorylation, homodimerization, and allosteric conformational changes. The activated complex then translocates to the nucleus where it recruits regulatory cofactors (coactivators), resulting in transactivation. Transactivation involves binding of the activated complex to a specific region of DNA, referred to as the estrogen response element (ERE), resulting in the synthesis of messenger RNA (mRNA) by a process called transcription. Afterwards, the mRNA translocates to the ribosomes in the cytoplasm where the genetic code is translated and specific proteins are synthesized. Transcription can also be inhibited by transrepression, in which the ER complex interacts with a corepressor protein, thereby altering recruitment of coactivators, and leading to gene-specific repression.

In contrast to genomic mechanisms of estrogen action, non-genomic mechanisms involve rapid estrogen signaling that takes place within seconds or minutes (11). This occurs via membrane-associated ERs or other cell surface receptors that can activate rapid downstream pathways, including kinase signaling.

Human genome-wide searches for high affinity EREs have demonstrated that they occur in many of the genes belonging to the procoagulant and anticoagulant pathways (12). They include the hepatic-specific coagulation factors II, V, VIII, IX, X, XI, and XII, as well as the anticoagulants protein S and protein C.

## Pharmacokinetics of estrogens

Since circulating estrogens affect concentrations of coagulation and fibrinolytic factors, it is important to know their pharmacokinetics, and how pharmacokinetic parameters differ among various estrogens. Much of our knowledge of estrogen pharmacokinetics is based on serum or plasma levels measured by immunoassay methods (13). Although radioimmunoassays (RIAs) with preceding purification steps have provided valuable data on the pharmacokinetics of estrogens, they lack the specificity and sensitivity of mass spectrometry (MS) assays, which are now considered the gold standard for steroid hormone measurements. In addition, direct immunoassays without a preceding purification step have been used in estrogen pharmacokinetic studies. Such assays can overestimate circulating estrogen levels grossly, making it difficult to establish accurate pharmacokinetic parameters (13). Nevertheless, a general idea of how pharmacokinetic parameters differ among the different estrogens used for contraception or HT can be obtained, as summarized below.

Two clinically important pharmacokinetic parameters of a drug are its bioavailability and half-life. The bioavailability of a drug is the extent to which it enters the systemic circulation after undergoing hepatic first-pass metabolism. The half-life of a drug is the time required for a drug’s blood level to fall to 50% of its maximal level. The bioavailability of E_4_ is high (14), which is likely due to its limited metabolism. In contrast, EE has a moderate bioavailability (on average, 40-45%) (15), and the bioavailability of E_2_ is very low (<2-10%) (16), due to its extensive hepatic first-pass metabolism. As for the bioavailability of E_2_ in CEEs, it is not determined since it comprises only 1-2% of the dose. Following oral administration of CEE, E_2_ is formed predominantly from estrone sulfate (E_1_S), the predominant component of CEE, during hepatic first pass metabolism.

In the circulation, most of the EE and E_4_ are loosely bound to albumin, whereas about 61% of E_2_ is bound to albumin and 37% with high affinity to sex hormone binding globulin (SHBG) (17). A small percentage (1-2%) of E_2_ is non-protein-bound (free). Only the free steroid fraction can enter cells and either undergo metabolism or exert biologic effects (free hormone hypothesis) (18). It is important to note that equine estrogens in CEEs also bind to SHBG and albumin in a manner similar to that of E_2_ (19).

The oral bioavailability of different estrogens depends on absorption within the digestive system. E_4_ is absorbed very rapidly and reaches a median time (t_max_) to reach maximum concentration (C_max_) of 0.25-0.5 h following dosing with 1, 10, or 100 mg E_4_ in postmenopausal women; the levels then fall sharply (14). EE is also absorbed rapidly, with maximum concentrations attained between 1–2 h following dosing with 0.03 mg EE in premenopausal women (20). In contrast, following oral administration of 1 or 2 mg E_2_, circulating E_2_ levels rise gradually and C_max_ is attained around 5 h; the levels are still elevated at 8 h (21). As for orally administered CEE (2 x 0.625 mg), the estrogenic components show slow absorption, with a t_max_ of 5–9 h (22).

Reported terminal half-lives (t_½_) for the different orally administered estrogens are: 5–30 h for EE (23); 13–20 h for E_2_ (24); and 28 h for E_4_ (25). For key metabolites of CEE, reported mean values include 17.1, 19.1, 11.5, and 13.3 h for E_1_, E_2_, equilin, and 17β-dihydroequilin, respectively (26).

## Potency of estrogens

The potency of an estrogen refers to its strength. It can be defined as a measure of the doses of 2 different drugs required to produce the same pharmacologic effect. The relative potencies of orally administered E_2_, CEE, and EE were determined in a study in which their effects on follicle-stimulating hormone (FSH) and estrogen-sensitive hepatic proteins (SHBG, CBG, and angiotensinogen) were evaluated in postmenopausal women who were treated with different doses of these estrogens (27). The results show that on a weight basis EE was by far the most potent estrogen. Compared to E_2_, the relative potency of EE was 614, 500, and 331 times greater with regard to the responses of SHBG, CBG, and angiotensinogen, respectively. In contrast, the relative potency of CEE compared to E_2_ was 3.2, 1.3, and 5 times greater with regard to the respective hepatic proteins. It is important to realize that ring B unsaturated estrogens such as equilin, equilenin, 17β-dihydroequilin, and 17β-dihydroequilenin are biologically active, and contribute to the estrogenicity of CEE. In fact, 17β-dihydroequilin has a higher relative binding affinity for ERα and ERβ than E_2_ (28). The pharmacologic effects of CEE are the result of the sum of the individual activities of its components (29).

The high estrogenic potency of EE is also evident in studies in which it was administered parenterally. Treatment of premenopausal women with a transdermal patch delivering 20 µg/d of EE and 150 µg/d of norelgestromin resulted in significant increases from baseline in serum levels of SHBG, CBG, TBG, and hs-CRP (30). The levels of SHBG and TBG were significantly greater than those associated with a combined oral contraceptive (COC) containing 35 µg EE/250 µg norgestimate, which was also administered in the same study. In addition, it has been shown that vaginal delivery of 15 µg EE combined with 150 µg of segesterone acetate from a contraceptive ring in premenopausal women did not reduce EE-associated increase in hepatic proteins (31). In contrast to these studies, transdermal E_2_ treatment in postmenopausal women has negligible effects on SHBG, CBG, and TBG (32).

As for E_4_, based on limited data it appears that this estrogen has a considerably lower impact on estrogen-sensitive hepatic proteins. In a study comparing the effect of a COC containing either E_4_ combined with drospirenone or EE in combination with drospirenone for 6 cycles, E_4_ combined with drospirenone had a significantly lower impact (+ 55%) on SHBG compared to the other formulation (+ 251%) (33).

## Effect of estrogens on hemostasis

The process of hemostasis involves several factors that act simultaneously to create a balance between coagulation and anticoagulation. Dysregulation of this system may increase the risk of arterial or venous thrombosis and subsequent tissue ischemia. Although estrogens are known to affect multiple hemostatic markers, the exact molecular mechanism of estrogen-induced thrombosis is not clear. However, it is possible that small changes in those markers by different types, routes, and/or doses of estrogens may increase the risk of thrombosis.

Most studies on the effect of estrogens on hemostasis have been carried out in premenopausal women using COCs; studies in postmenopausal women using HT are limited. The studies with COCs have been carried out predominantly in combination with a progestin. Most of the studies have involved EE-based COCs and considerably fewer studies exist with E_2_-based COCs. The effects of E_4_-based COCs on hemostasis and risk for thrombosis have been studied only recently, and long-term large-scale studies are still lacking. Also, limited data on the effects of oral E_2_ and CEE, and transdermal E_2_ used for menopausal HT, on hemostatic parameters exist. Nevertheless, some conclusions can be drawn from those studies regarding how the estrogens compare with respect to their effects on coagulation and fibrinolysis. Studies related to the effect of estrogens on hemostasis among COC and menopausal HT users are discussed separately.

## Estrogens and hemostasis in COC users

EE is most commonly used in COCs and has been shown to have a dose-dependent effect on thrombosis risk (34). These findings have led to a decrease in prescribed dosages and exploration of alternative estrogen formulations for oral contraception. Agren and coworkers (35) compared the effects of 6 cycles of either E_2_ (1.5 mg)/nomegestrol acetate (NOMAC) (2.5 mg) or EE (30 µg)/levonorgestrel (LNG) (150 µg) on hemostatic parameters in 121 premenopausal women. The COC with LNG was chosen because LNG is able to partially counteract the effects of EE on coagulation. Minimal changes from baseline to the end of either treatment were found in the 2 groups in prothrombin, activated factor VII, coagulated activated factor VII, and factor VIII. However, there were significant differences between the 2 groups in the anticoagulant indicators antithrombin III, total protein S, and protein C, and minor differences in the activated partial thromboplastin time (aPTT)-based activated protein C (APC) sensitivity ratio and in free protein S. The EE/LNG group had a substantial significant increase from baseline in the endogenous thrombin potential (ETP)-based APC sensitivity ratio. There was also a small increase in the prothrombin fragment 1 + 2 (F1 + 2) in the EE/LNG group but not in the E_2_/NOMAC group, whereas D-dimer did not change in either group. While CRP levels remained within the normal range of values for premenopausal women, there was a greater percent change from baseline in the EE/LNG group.

Another double-blind, randomized study by Gaussem and coworkers (36) compared a lower dose of EE (20 µg)/LNG (100 µg) to E_2_ (1.5 mg)/NOMAC (2.5 mg) among healthy reproductive aged women in 3 consecutive cycles. Most of the differences in measured coagulation and fibrinolysis parameters between the 2 groups were relatively small, and values at the end of treatment were within normal ranges.

A randomized, open-label, single-center study by Junge and coworkers reported a comparison of a multiphasic formulation combining E_2_V (1–3 mg) and dienogest (DNG) (2–3 mg) with a triphasic formulation combining EE (30-40 µg) and LNG (50-125 µg) among 60 healthy reproductive aged women over a period of 7 cycles (37). Coagulation and fibrinolytic parameter values from baseline to end of treatment showed relatively small changes and remained within normal ranges in both groups.

These short-term studies comparing hemostatic parameters in women receiving either E_2_ or EE, each combined with a different progestin, appear to indicate that the E_2_- and EE-based COCs generally have minimal effects on most coagulation and fibrinolytic markers, and weaker effects on some markers with E2.

Recently, E_4_ has gained interest as an option for oral contraception with a neutral effect on hemostatic parameters. In a randomized open-label exploratory study, Douxfils and coworkers (33) assessed hemostatic effects of an E_4_-based COC compared to 2 different EE-based COCs. Thirty-nine premenopausal women received E_4_ (15 mg)/DRSP (3 mg), 30 women received EE (30 µg)/LNG (150 µg), and 32 women received EE (20 µg)/DRSP (3 mg) for six 28-day cycles. Median changes in the coagulation factors fibrinogen, prothrombin, factor VII, factor VIII, and Von Willebrand factor were generally small, and not significantly different between the 3 groups, with the exception of factor VII which was significantly increased with EE/DRSP compared to the other 2 formulations. There was a 30% median change in the ETP-based APC sensitivity resistance at cycle 6 in the women receiving E_4_/DRSP, which was significantly lower than in those receiving EE/LNG (105%) or EE/DRSP (219%). There were also small changes from baseline to end of treatment in the anticoagulant parameters antithrombin, protein S activity, free protein S, protein C, and the tissue pathway inhibitor (TFPI) with E_4_/DRSP and EE/LNG. Changes in protein S activity, free protein S, and protein C were significantly greater in the EE/DRSP group compared to the E_4_/DRSP and EE/LNG groups. Assessment of fibrinolytic markers (plasminogen, plasminogen activator inhibitor, tissue plasminogen activator) indicated a weak impact of E_4_/DRSP, but any potential hypo-fibrinolytic or hyper-fibrinolytic profile of E_4_/DRSP impacting VTE risk could not be determined from these data.

Morimont and coworkers (38) conducted a randomized, open-label parallel study to compare the effects of E_4_ (15 mg)/DRSP (3 mg) to EE (20 µg)/DRSP (3 mg) and EE (30 µg)/LNG (150 µg) on thrombin generation over 6 treatment cycles in a cohort of women. They concluded that E_4_/DRSP had no impact on thrombin generation measured by lag time, peak, time to peak, ETP, and mean velocity rate index, whereas the EE-containing COCs were associated with a shift to a prothrombotic state.

According to data from the above short-term comparative studies of the effects of different types of estrogens on hemostatic parameters, the E_2_- and EE-based COCs generally have similar effects on coagulation and fibrinolytic markers while COCs containing E_4_ seem to have less impact on hemostasis, as summarized in **Table 1**.

**Table 1:** Summary of studies showing the effects of ethinylestradiol-, estradiol-, and estetrol-based combined oral contraceptives on the coagulation and fibrinolytic systems in premenopausal women.

Study	Percentage change from baseline (%)								
	Agren et al. (35) (median change)		Junge et al. (37) (intraindividual change)		Douxfils et al. (33) (median change)			Gaussem et al. (36) (median change)	
	E_2_/NOMAC	EE/LNG	E_2_V/DNG	EE/LNG	E_4_/DRSP	EE/LNG	EE/DRSP	E_2_/NOMAC	EE/LNG
Anticoagulant proteins									
Antithrombin III	3.9	-3.6^*^	0.8	-3.0	-1.0	-5.0	-3.5	0.3	-4.4^*^
Protein S activity			1.8	-11.7^*^	-4.0	-5.0	-30.5^*^		
Protein S free	13.3	11.9			5.0	-3.0	-22.5^*^		
Total protein S	4.7	-3.6^*^							
Protein C	-3.1	8.2^*^			2.0	7.0	17.5^*^		
Protein C activity			8.3	14.5					
TFPI free					-8.5	1.0	-20.0		
Fibrinolytic proteins									
Plasminogen					12.0	40.0^*^	35.5^*^	6	30^*^
PAI-1			-10.6	-36.2	20.0	0.0	0.0	-3.1	-8.0^*^
t-PA			-3.7	-5.1	-7.0	-33.0^*^	-39.5^*^		
Marker for ongoing coagulation									
D-dimer	0.0	0.0	-2.1	62.9^*^	4.0	7.0	0.0	-53	43^*^
Prothrombin fragment 1 + 2	-1.7	13.5	-0.6	117.3	23.0	71.0^*^	64.0^*^	-0.02	0.08^*^
Procoagulant factors									
Fibrinogen			7.9	28.1^*^	10.0	5.0	16.0		
Prothrombin/Factor II	-0.9	3.0			7.0	13.0	7.0		
Factor VII					-3.0	-5.0	20.0^*^		
Factor VIIa	8.8	14.4							
Factor VIIc	1.0	-12.7^*^							
Factor VII activity			13.5	24.4^*^					
Factor VIII	4.8	6.8			5.0	3.0	9.0		
Factor VIII activity			6.9	7.5					
Von Willebrand factor					5.0	-2.0	13.0		
Functional coagulation tests									
nAPCsr					30.0	164.5^*^	218.5^*^		
APC resistance (aPTT)	3.3	2.0	-5.3	-7.0					
ETP based APCr	60.0	146.4^*^							

APC, activated protein C; DNG, dienogest; DRSP, drospirenone; E_2_, estradiol; E_2_V, estradiol valerate; ETP, endogenous thrombin potential; LNG, levonorgestrel; nAPCr, normalized APC sensitivity ratio; NOMAC, nomegestrol acetate; PAI-1, plasminogen activator inhibitor-1; TFPI, tissue factor pathway inhibitor; t-PA, tissue plasminogen activator.

*Statistically significant difference (P<0.005) from the reference product (reference products are E_2_/NOMAC for Agren et al. (35); E_2_V/DNG for Junge et al. (37); E_4_/DRSP for Douxfils et al. (33); E_2_/NOMAC for Gaussem et al. (36).

Adapted with permission from Stanczyk et al. (4), licensed under License #6102080366944, Elsevier.

In the absence of standardized assay methods to evaluate coagulability status, there has been misinterpretation of data obtained with 2 activated protein C (APC) sensitivity assays, which quantify the effects of APC on the aPTT and ETP (39). While the aPTT-based APC resistance assay measures the time it takes for blood to clot with and without the addition of APC to reflect reduced sensitivity to APC, the ETP-based APC resistance assay measures the total amount of thrombin generated in the blood sample over time with and without addition of APC. The aPTT assay is highly influenced by prothrombin and factor VIII levels, while the ETP assay is more sensitive to free protein S and free TFPI levels (39, 40). As the latter factors are much more influenced by the former ones, it may in part explain the inconsistent results between the 2 functional APC assays (39). In addition, the normalized APC sensitivity ratio (nAPCsr) has been shown to predict the risk of VTE where this ratio increased (41). Previous studies using this nAPCsr support that E_4_/DRSP may be correlated with lower VTE risk compared to EE/LNG and EE/DSG. This evidence suggests the use of the ETP-based APC resistance assay to identify VTE risk among women who are taking estrogen (39, 40, 42, 43). APC resistance is identified among individuals with factor V Leiden, the most common hereditary thrombophilia. APC resistance also occurs with other hereditary (some rare F5 variant) and acquired causes (including hormone-induced, solid tumor, hematologic malignancies). The International Society on Thrombosis and Haemostasis Scientific and Standardisation Committee recommends the ETP-based APC resistance to detect the acquired APC resistance induced by COCs; in particular, the current aPTT-based assays are only sensitive towards factor V Leiden mutation due to the introduction of deficient plasma in the test (44).

## Estrogens and hemostasis in menopausal HT users

Several studies quantified hemostasis in order to examine the association of estrogen used in menopausal HT with thrombosis risk. A meta-analysis of 48 studies including 40–68 years old postmenopausal women (6,229 HT users and 24,974 non-users) explored the association between HT use on coagulation factors (45). The study concluded that HT was associated with significantly decreased fibrinogen, factors VII, antithrombin, protein C, protein S and significantly increased plasminogen levels.

When comparing different type of estrogen, Blondon and coworkers reported from a retrospective cohort study of 140 postmenopausal women that women using E_2_ had significant lower thrombin generation and higher total protein S, which suggests less prothrombotic status compared to CEE (46). Studies on the effect of E_4_ use in menopausal HT on hemostasis are lacking.

Routes of estrogen administration for menopausal HT have shown different hemostasis effects. A double-blind placebo-controlled study among 152 menopausal women randomized to oral E_2_ (1 mg), oral E_2_ (1 mg) with gestodene (25 µg), transdermal E_2_ (50 µg), or placebo showed a significant increase in nAPCsr in all treatment groups (47). It is important to note that the increase in nAPCsr was significantly higher in the oral compared to transdermal E_2_ group. While there were significant changes in fibrinogen, factor VII, thrombin-antithrombin III complexes, tissue-type plasminogen activator and D-dimer with oral E_2_, no significant changes of these markers were identified in the transdermal E_2_ group (48). The data suggest that transdermal estrogen has a minor effect on hemostasis compared to oral estrogen.

To explore the effect of estrogen doses on hemostasis, Lobo and coworkers (49) measured hemostatic factors in 749 postmenopausal women who were randomized to different doses of CEE (0.625 mg, 0.45 mg, or 0.3 mg) with and without MPA for one year. Overall, CEE was found to increase plasminogen activity and decrease plasminogen activator inhibitor 1 activity, antithrombin III activity, and protein S. The study suggests that lower doses of CEE tend to induce more favorable changes in hemostasis markers towards lower risk of thrombosis. Regarding doses of E_2_ and hemostasis, Eilertsen and coworkers (50) conducted a randomized open-label, comparative study of E_2_ (2 mg)/norethisterone acetate (NETA) (1 mg), E_2_ (1 mg)/NETA (0.5 mg) among 202 menopausal women. D-dimer increased markedly in the conventional-dose HT group but remained unchanged in the low-dose HT group. The reductions in both clotting factors and inhibitors were markedly more pronounced in the conventional-dose HT group compared to the low-dose HT group. The study suggests that low-dose HT is associated with less activation of coagulation than conventional dose HT.

To determine whether biomarkers of thrombosis can identify menopausal women at risk of VTE, a nested case-control study (51) was carried out using the Women’s Health Initiative trial data (52, 53). From the total of 27,347 menopausal women randomized to treatment with CEE with or without MPA or placebo, 215 women who developed thrombosis and 867 women without thrombosis at 1 year were included in the study. The study found that women with thrombosis had lower protein C and free protein S, and higher D-dimer, prothrombin fragment 1 + 2, and PAP. Among these markers, D-dimer was most strongly related to VTE (OR, 6.0; 95% CI, 3.6-9.8). From these findings, the study suggested the potential for clinical use of D-dimer testing to evaluate the risk for thrombosis in menopausal women before prescribing HT.

## Effect of estrogens on thrombosis

The increased procoagulant factors and decreased anticoagulatory mechanisms observed with estrogen use have been linked with an increased risk of thrombosis, this includes both venous and arterial thrombosis. The most frequent clinical sign of estrogen-related thrombosis is VTE, affecting the deep veins of the legs or the pulmonary arteries, typically within the first few months of use. Estrogen has also been associated with an increased risk of thrombosis at uncommon sites and with arterial thrombosis (34). The association between estrogens and thrombosis among COC and menopausal HT users is discussed separately.

## Estrogens and Thrombosis in COC Users

Recently, there has been an increasing interest in how the types and delivery routes of estrogen influence the risk of VTE. Some studies have proposed that non-oral routes of administration may result in lower serum estrogen concentrations and fewer systemic effects than oral administration. We summarize the studies related to estrogens used for contraception on risk for thrombosis in **Table 2**.

**Table 2:** Effect of estrogens on risk for thrombosis in women using combined oral contraceptives; summary from systematic review.

Study	Reference	Results
Type of estrogen	Morimont et al. (40)	EE-based: 5–12 per 20,000 women-years
		E_2_-based: 2–7 per 10,000 women-years
	Douxfils et al. (54)	E_2_-based compared to EE-based: Pooled OR 0.67 (95% CI, 0.51–0.87)
	Bauerfeind et al. (55)	E_2_V/DNG compared to EE/LNG: Adjusted HR 0.46 (95% CI, 0.22–0.98)
Route of estrogen administration	Tepper et al. (56)	Patch vs. oral: 2 studies with significant increased risk; 1 study with non-significant increased risk; 4 studies with no increased risk Vaginal ring vs. oral: 1 study with significant increased risk; 2 studies with no increased risk
Estrogen dosage	De Bastos et al. (57), Oedingen et al. (58)	Higher EE dose is associated with higher risk.

### Type of estrogen

The natural estrogens, E_2_ and E_4_, have been compared to the synthetic estrogen, EE, with respect to their effects on thrombosis. A review by Morimont (40) summarized 7 studies that compared COCs containing natural estrogens (E_2_, E_2_V, E_4_) versus synthetic EE on VTE risk. The study estimated VTE risk as 2/10,000 women-years (total years that women were followed up in the study) for non-pregnant, non-COC-users. The estimated VTE risk for EE-based COC users ranged from 5-12/20,000 women-years depending on the progestin type (59), whereas the estimated VTE risk in E_2_-based COC users ranged from 2-7/10,000 women years (60, 61). From these results, the study suggested that natural estrogens may offer improved cardiovascular safety.

Douxfils and coworkers (54) performed a pooled analysis of 5 large observational studies containing over 560,000 women aged 18 and older to compare E_2_-based COCs (72,210 women) to EE-based COCs (487,942 women). VTE events were identified among 59 women using E_2_ and 685 women using EE. Each individual study reported odds ratios (OR) comparing VTE risk between the 2 groups: OR, 1.77; 95% CI, 0.59–5.35 (62), OR, 0.57; 95% CI, 0.37–0.87 (63), OR, 0.63; 95% CI, 0.33–1.20 (61), OR, 0.67; 95% CI, 0.34–1.33 (64), and OR, 0.70; 95% CI, 0.41–1.20 (55). While most individual studies did not reach statistical significance, the pooled analysis showed a statistically significant 33% lower risk of VTE in users of natural E_2_-based COCs compared to synthetic EE-based COCs (pooled OR, 0.67; 95% CI, 0.51–0.87).

A recent pooled analysis by Bauerfeind and coworkers (55) indicated that the use of E_2_V–dienogest (E_2_V/DNG) is linked to a significantly lower VTE risk compared to the EE/LNG combination, despite the overall low incidence of VTE in both groups. The analysis reported a 54% lower VTE risk among E_2_V/DNG users compared to EE/LNG users with a propensity score-adjusted hazard ratio (HR) of 0.46 (95% CI, 0.22–0.98). Subgroup analysis from the European cohort reflected similar trends, showing a propensity score–adjusted HR of 0.40 (95% CI, 0.18–0.89), which corresponds to a significant 60% lower risk of VTE (55). These findings suggest that E_2_V/DNG may be a safer option than EE/LNG for women who are at higher risk for thromboembolic events.

A new prolonged-release formulation containing EE (20 µg)/DNG (2 mg) has recently been released and offers high contraceptive efficacy. The fluctuations of EE concentrations at steady state have been shown to be significantly lower as compared to the traditional EE (30 µg)/DNG (2 mg) formulation (65). The comparative pharmacokinetic data show that with the new formulation the C_max_ of EE is reduced by 52% with approximately a 3-fold increase in T_max_. In the same comparison, the C_max_ of DNG was reduced by 21% and T_max_ was increased by about the same amount as EE. As for the AUC of EE, it was reduced by 34%, but there was little change in the AUC of DNG between the 2 formulations. Minimizing the exposure of EE is desirable because it reduces estrogen-related adverse effects.

A major concern in the prolonged-release EE/DNG phase 3 studies was that there were a total of 8 participants who experienced VTE (65). The apparent high rate of VTE in the phase 3 studies with the prolonged-release formulation was accepted as a chance finding, and it was considered to be possibly due to inclusion of study participants for which COC use should have been contraindicated. Users of the prolonged-release formulation will be closely monitored. A recent study showed that the new EE/DNG formulation was not associated with any meaningful changes in analyzed coagulation and fibrinolytic parameters, indicating that this formulation does not have an impact on these parameters (66).

Currently, E_4_-based COCs, such as those that include E_4_/DRSP, are undergoing evaluation for the risk of VTE in post-marketing studies. So far, a low number of thrombotic events have been reported, which is consistent with findings from phase 3 trials indicating low rates of VTE. However, there is a lack of direct comparative studies with products containing EE. These findings collectively suggest that natural estrogens, particularly E_2_ and E_4_, should be reconsidered for inclusion in COC formulations, as they may improve cardiovascular safety profiles for women of reproductive age (54).

In terms of progestin type and VTE risk, combination with androgenic progestins such as LNG and norethindrone has been suggested to antagonize the EE-associated risk of VTE to a greater extent than combination with non-androgenic progestins such as desogestrel (67–70). However, after correcting for confounding factors such as weight, smoking status, alcohol use, age, and duration of use, other studies have found no difference in VTE risk between androgenic and non-androgenic progestins (71–74).

### Route of estrogen administration

A cohort study of 1.6 million women reported that the adjusted relative risk (RR) of VTE in patch users was 2.3 (95% CI, 1.0-5.2) and in vaginal ring users it was 1.9 (95% CI, 1.3-2.7) compared with users of COCs containing LNG (75). Another cohort study comparing thrombosis risk between COC versus vaginal ring users revealed that after 66,489 women-years of follow up VTE incidence was 9.2 vs. 8.3 per 10,000 women-years in COC users compared to vaginal ring users with an adjusted HR of 0.8 (95% CI, 0.5-1.5), and the authors concluded that COC and vaginal ring use was associated with a similar VTE risk (76).

A systematic review of 6 studies comparing thrombosis risk between COC versus transdermal patch users demonstrated an inconsistent VTE risk as 2 studies found a statistically significantly elevated risk among patch users (risk estimates, 2.2-2.3); one found an elevated risk that did not meet statistical significance (risk estimate, 2.0), and 4 found no increased risk (56). In the same systematic review to compare the VTE risk between vaginal ring vs. COC users, one study found a statistically significantly elevated risk among patch users (risk estimate 1.9) while 2 studies did not find a significant association (56).

In summary, there is still inconsistent evidence to conclude whether or not there is an association between estrogen route and thrombosis risk.

### Estrogen dosage

Estrogen dosage plays a role in VTE risk, as EE administration has been shown to increase thrombosis risk in a dose-dependent manner (77, 78). Low-dose COCs containing <50 µg of EE have been used in an attempt to reduce the risk of VTE, but the effects on coagulation even with very low EE doses remain present. Women with known cardiovascular risk factors, including smoking, high blood pressure, diabetes, obesity, age, history of thrombosis or other coagulation abnormalities, are at highest VTE risk from COC use (79–82).

Two systematic reviews and meta-analyses clearly suggested that higher estrogen doses are associated with increased thrombosis risk (57, 58). A dose related effect of EE was observed for gestodene, desogestrel, and levonorgestrel, with higher doses being associated with higher thrombosis risk. These results suggested prescribing the lowest possible EE dose to avoid VTE.

### Estrogens and Thrombosis in Menopausal HT Users

Regarding HT, the number of studies examining VTE risk are fewer. The Women’s Health Initiative trial showed an increased risk of VTE in both the CEE and CEE/MPA groups, suggesting a need for alternative options for HT (52, 53). Low-dose EE has been proposed for the treatment of menopausal symptoms; however, it carries an increased risk of VTE as with EE-based COCs. Both oral and transdermal E_2_ have been suggested to have a smaller impact on VTE risk; however, the results have been mixed. Overall, vaginal formulations to treat genitourinary symptoms of menopause, including vaginal dryness, dyspareunia, and problems with urination, are associated with low systemic hormone levels. This suggests that VTE risk is low with vaginal estrogens; however, more studies are needed to study the exact effects of the vaginal application. We summarize the studies related to estrogens used for menopausal HT on risk for thrombosis in **Table 3**.

**Table 3:** Effect of estrogens on risk for thrombosis in women using menopausal hormone therapy; summary from systematic review.

Study	Reference	Results
Type of estrogen	Smith et al. (83)	CEE vs. E_2_: CEE had higher VTE risk (OR, 2.08; 95% CI, 1.02-4.27)
	Vinogradova et al. (84)	E_2_ vs. CEE: E_2_ only had lower VTE risk (OR, 0.85; 95% CI, 0.76-0.95) E_2_ plus progestin had lower VTE risk (OR, 0.83; 95% CI, 0.76-0.91)
	Blondon et al. (85)	E_2_ vs. CEE: Oral E_2_ had no significant VTE risk (OR, 0.96; 95% CI, 0.64-1.46) Transdermal E_2_ had no significant VTE risk (OR, 0.95; 95% CI, 0.60-1.49)
Route of estrogen administration	Rovinski et al. (86)	Oral vs. non-oral HT: Overall oral HT increased risk (OR, 1.72; 95% CI, 1.47-2.01) Estrogen only (OR, 1.43; 95% CI, 1.34-1.53) Estrogen plus progestin (OR, 2.35; 95% CI, 1.9-2.9).
Estrogen dosage	Binkowska et al. (87)	Low dose estrogen: (OR, 1.57; 95% CI, 1.48-1.68) High dose estrogen: (OR, 1.91; 95% CI, 1.70-2.16) Different risk by dose (p = 0.004)
Timing of estrogen initiation	Salpeter et al. (88)	Reduced risk when less than 60 years old (OR, 0.68; 95% CI, 0.48-0.96) No significant risk when older than 60 years old (OR, 1.03; 95% CI, 0.91-1.16).
	Boardman et al. (89)	Reduced risk in younger menopausal women (OR, 0.52; 95% CI, 0.29-0.96) No effect in older menopausal women (OR, 1.07; 95% CI, 0.96-1.20).

### Type of estrogen

Different estrogen and different progestin types used in menopausal HT have been reported to be associated with risk of thrombosis differently. The Women’s Health Initiative trials reported that CEE increase the risk of VTE (52). CEE 0.625 mg/d plus MPA 2.5 mg/d increased thrombosis risk significantly with a HR of 2.11 (95% CI, 1.26-3.55) when compared to placebo. CEE 0.625 mg/d alone increased thrombosis risk with a HR of 2.06 (95% CI, 1.57–2.70) compared to placebo (90). Several studies compared the thrombosis risk between CEE and E_2_ used in menopausal women, but the results were not consistent. A population-based case-control study of 68 VTE cases with 201 matched controls showed that CEE had a higher VTE risk (OR, 2.08; 95% CI, 1.02-4.27; P = 0.045) (83). Another nested case-control study of 80,396 women showed that compared to CEE, E_2_ only (OR, 0.85; 95% CI, 0.76-0.95) and E_2_ plus progestin (OR, 0.83; 95% CI, 0.76-0.91) had a lower VTE risk (84). In contrast, a retrospective cohort study among 51,571 HT users with a mean age of 54 years showed no significant VTE risk between oral E_2_ (OR, 0.96; 95% CI, 0.64-1.46) and transdermal E_2_ (OR, 0.95; 95% CI, 0.60-1.49) compared to CEE (85).

Regarding the type of progestogen used in HT several studies demonstrated different VTE risks. A cohort study with over 1 million postmenopausal women (91) revealed that oral combined estrogen-progestin therapy carried the highest risk of VTE, particularly when MPA was used. Specifically, women taking oral estrogen-progestin therapy with MPA had a RR of 2.67 (95% CI, 2.25–3.17), compared to a lower RR of 1.91 (95% CI, 1.69–2.17) for those using NET or LNG, which are considered androgenic progestins. These findings support the idea that not all progestins carry the same thrombotic risk, with MPA being prothrombotic. The study confirmed that using transdermal estrogen-only therapy, which avoids the hepatic first pass and reduces blood clot risks, does not increase the chances of VTE. The RR was 0.82, with a confidence interval of 0.64 to 1.06. Additionally, the risk is highest during the first 2 years of HT. This shows that it is important to monitor patients closely during the early stages of treatment. These results match earlier research by Canonico and coworkers (92, 93). They found that progestins such as micronized progesterone and pregnane derivatives do not increase the risk of VTE. However, norpregnane derivatives do raise the risk of VTE significantly. This emphasizes the importance of choosing the right estrogen and progestogen in HT to lower the chances of blood clots.

### Route of estrogen administration

Most studies of VTE risk in estrogen users have been carried out using oral estrogen. However, since it was shown that transdermal E_2_ has little or no effect on markers of coagulation and fibrinolysis (86), there was interest in studying the effects of the transdermal E_2_ route on VTE risk (94, 95).

A cohort study (ESTHER) (92, 93), which followed over 80,000 postmenopausal women over 10 years, with 549 VTE cases reported in women using oral estrogen, was associated with increased VTE (HR, 1.7; 95% CI, 1.1–2.8), while transdermal estrogen showed no significant association with VTE (HR, 1.1; 95% CI, 0.8–1.8). Likewise, another case-control study of 271 cases and 610 controls also reported that oral estrogen had an increased VTE risk (OR, 4.2; 95% CI, 1.5–11.6) whereas transdermal estrogen was not associated with VTE (OR, 0.9; 95% CI, 0.4–2.1). In a multicenter, hospital-based study, postmenopausal women (155 cases and 351 matched controls) were treated either transdermally (patch or gel containing ≤50 µg E_2_) or orally with a mean dose of 1.5 mg E_2_ (96). The study reported that current users of oral estrogen were at increased risk of VTE, whereas there was no association between VTE risk and use of transdermal E_2_. These findings consistently suggest that transdermal estrogen may be a safer option for HT regarding the risk of thrombosis.

An updated meta-analysis of data on VTE risk among HT users provided further evidence that oral but not transdermal estrogens increase VTE risk (97). However, a more recent retrospective cohort study that included postmenopausal veterans who used either oral CEE (N=38,421) or E_2_ (N=6,501), or transdermal E_2_ (N=6.649) did not confirm the previously observed difference between oral vs. transdermal estrogen (85).

A meta-analysis by Rovinski and coworkers (86) compared the effects of oral and non-oral (mainly transdermal) HT on VTE risk in subjects with no history of VTE. The meta-analysis reviewed a total of 22 studies, which included 9 case-control studies, 9 cohort studies, and 4 randomized controlled trials. Among these 22 studies, 113,059 women used non-oral HT, 281,018 used oral HT, and 868,514 were in the control group. The comparison of oral HT with non-oral HT and the control group revealed that oral HT led to a significant increase in VTE risk (OR, 1.72; 95% CI, 1.47-2.01]), both in the estrogen only group (OR, 1.43; 95% CI, 1.34-1.53) and the estrogen plus progestin group (OR, 2.35; 95% CI, 1.9-2.9). There was no change in VTE risk with non-oral HT use, including the estrogen only and estrogen plus progestin treatment groups. Notably, this study did not differentiate between different estrogen formulations or dosages prescribed. Based on these results, the route of estrogen administration does significantly impact VTE risk, likely due to differences in effects on hepatic coagulation factors. While this study adds to the discussion regarding the increased risk of VTE when estrogen is combined with a progestin, more research is needed to examine the actions of different progestogens individually.

Overall, the risk of VTE during HT mainly depends on how the estrogen is given. Oral HT, whether alone or with progestin, has been linked to a higher risk of VTE. In contrast, most studies show that transdermal and vaginal forms, which avoid hepatic first-pass metabolism, usually do not increase VTE risk. Among these methods, vaginal estrogen seems to have the lowest risk because it is absorbed minimally into the bloodstream. However, some recent studies have shown mixed results.

### Estrogen dosage

Another important factor affecting the risk of VTE due to HT in postmenopausal women is the dosage. Higher doses of estrogen have consistently been associated with more significant changes in coagulation markers and a heightened thrombotic risk. A meta-analysis of 6 observational studies evaluating the association between HT dose and VTE risk showed that the VTE risk was lower for low-dose estrogen; (low-dose estrogen OR, 1.57; 95% CI, 1.48-1.68) vs. high-dose estrogen (OR, 1.91; 95% CI, 1.70-2.16; *p* = 0.004) (87). A study by Sriprasert and coworkers (98) looked at how different doses of oral E_2_ and progesterone affected 1,512 postmenopausal women. The study found that higher doses of E_2_, especially 1 mg, led to shorter prothrombin time (PT) and aPTT. Participants also had lower levels of natural anticoagulants like antithrombin, protein C, and protein S. These changes suggest a higher risk for thrombosis that increases with higher doses of E_2_ and blood levels of E_2_, especially in women who are in late postmenopause.

## Timing of HT initiation

For VTE, a systematic review and meta-analysis of 19 clinical trials among 40,410 postmenopausal women (89) reported a similar VTE risk when menopausal HT was initiated within 10 years versus 10 years or more since menopause. Among postmenopausal women within 10 years since menopause, the pooled VTE risk was 1.74 (95% CI, 1.11–2.73) and among those who were 10 years or more since menopause, the pooled VTE risk was 1.96 (95% CI, 1.37–2.80).

For risk of arterial thrombosis, most importantly coronary heart disease (CHD), the timing of HT initiation was associated with the risk. A pooled analysis of 23 clinical trials among 39,049 menopausal women with 191,340 women-years of follow-up (88), showed a different CHD risk in younger versus older menopausal women. While HT significantly reduced CHD in menopausal women less than 60 years old (OR, 0.68; 95% CI, 0.48-0.96), HT had no significant association with CHD in menopausal women older than 60 years old (OR, 1.03; 95% CI, 0.91-1.16). Another meta-analysis of 19 trials with a total of 40,410 menopausal women (89) reported consistent findings of a significant reduction of CHD in younger menopausal women (OR, 0.52; 95% CI, 0.29-0.96) and no effect of HT on CHD in older menopausal women (OR, 1.07; 95% CI, 0.96-1.20).

## Conclusions

This review summarizes the mechanism of action, pharmacokinetics, and potency of estrogen to explain the effect of estrogen on hemostasis and risk of thrombosis. Estrogen affects changes in hemostasis that involve complex coagulation, anticoagulation and fibrinolysis cascades, and could explain the estrogen effect on thrombosis. The association of estrogen on thrombosis risk when used for contraception and menopausal HT among healthy women varies by type of estrogen, estrogen dosage, and route of estrogen administration.
